# Comparison of 454-ESTs from *Huperzia serrata *and *Phlegmariurus carinatus *reveals putative genes involved in lycopodium alkaloid biosynthesis and developmental regulation

**DOI:** 10.1186/1471-2229-10-209

**Published:** 2010-09-21

**Authors:** Hongmei Luo, Ying Li, Chao Sun, Qiong Wu, Jingyuan Song, Yongzhen Sun, André Steinmetz, Shilin Chen

**Affiliations:** 1 Institute of Medicinal Plant Development (IMPLAD), Chinese Academy of Medical Sciences & Peking Union Medical College, No.151, Malianwa North Road, HaiDian District, Beijing 100193, China; 2 Centre de Recherche Public-Santé, Luxembourg, L-1526 Luxembourg

## Abstract

**Background:**

Plants of the *Huperziaceae *family, which comprise the two genera *Huperzia *and *Phlegmariurus*, produce various types of lycopodium alkaloids that are used to treat a number of human ailments, such as contusions, swellings and strains. Huperzine A, which belongs to the lycodine type of lycopodium alkaloids, has been used as an anti-Alzheimer's disease drug candidate. Despite their medical importance, little genomic or transcriptomic data are available for the members of this family. We used massive parallel pyrosequencing on the Roche 454-GS FLX Titanium platform to generate a substantial EST dataset for *Huperzia serrata *(*H. serrata*) and *Phlegmariurus carinatus *(*P. carinatus*) as representative members of the *Huperzia *and *Phlegmariurus *genera, respectively. *H. serrata *and *P. carinatus *are important plants for research on the biosynthesis of lycopodium alkaloids. We focused on gene discovery in the areas of bioactive compound biosynthesis and transcriptional regulation as well as genetic marker detection in these species.

**Results:**

For *H. serrata*, 36,763 unique putative transcripts were generated from 140,930 reads totaling over 57,028,559 base pairs; for *P. carinatus*, 31,812 unique putative transcripts were generated from 79,920 reads totaling over 30,498,684 base pairs. Using BLASTX searches of public databases, 16,274 (44.3%) unique putative transcripts from *H. serrata *and 14,070 (44.2%) from *P. carinatus *were assigned to at least one protein. Gene Ontology (GO) and Kyoto Encyclopedia of Genes and Genomes (KEGG) orthology annotations revealed that the functions of the unique putative transcripts from these two species cover a similarly broad set of molecular functions, biological processes and biochemical pathways.

In particular, a total of 20 *H. serrata *candidate cytochrome P450 genes, which are more abundant in leaves than in roots and might be involved in lycopodium alkaloid biosynthesis, were found based on the comparison of *H. serrata *and *P. carinatus *454-ESTs and real-time PCR analysis. Four unique putative CYP450 transcripts (Hs01891, Hs04010, Hs13557 and Hs00093) which are the most likely to be involved in the biosynthesis of lycopodium alkaloids were selected based on a phylogenetic analysis. Approximately 115 *H. serrata *and 98 *P. carinatus *unique putative transcripts associated with the biosynthesis of triterpenoids, alkaloids and flavones/flavonoids were located in the 454-EST datasets. Transcripts related to phytohormone biosynthesis and signal transduction as well as transcription factors were also obtained. In addition, we discovered 2,729 and 1,573 potential SSR-motif microsatellite loci in the *H. serrata *and *P. carinatus *454-ESTs, respectively.

**Conclusions:**

The 454-EST resource allowed for the first large-scale acquisition of ESTs from *H. serrata *and *P. carinatus*, which are representative members of the *Huperziaceae *family. We discovered many genes likely to be involved in the biosynthesis of bioactive compounds and transcriptional regulation as well as a large number of potential microsatellite markers. These results constitute an essential resource for understanding the molecular basis of developmental regulation and secondary metabolite biosynthesis (especially that of lycopodium alkaloids) in the *Huperziaceae*, and they provide an overview of the genetic diversity of this family.

## Background

The *Huperziaceae *consist of two genera, *Huperzia *and *Phlegmariurus*, with a total of about 150species worldwide [1,2]. The *Huperziaceae *grow very slowly, normally requiring fifteen to twenty years of growth from spore germination to maturity [3,4]. Many studies have investigated the natural products in *Huperziaceae *plants, including lycopodium alkaloids, triterpenes, flavones and phenolic acids, some of which possess pharmacological activities [5-8]. Among these compounds, the lycopodium alkaloids, especially huperzine A (Hup A), were originally isolated from *Huperzia serrata *and have been investigated extensively and intensively [2-4,7].

*Huperzia serrata *(Thunb.) Trev. is a member of the *Huperzia *genus. *Phlegmariurus carinatus *(Desv.) Ching, also known as club moss, is a member of the *Phlegmariurus *genus [9]. *H. serrata *and *P. carinatus *are good candidate model plants for studying the biosynthetic pathways of lycopodium alkaloids since they accumulate various types of lycopodium alkaloids including lycopodines, lycodines and fawcettimines, some of which are valuable for pharmaceutical applications [2,4]. In particular, Hup A, which belongs to the lycodines, has been used not only as an anti-Alzheimer's disease (anti-AD) drug candidate in China due to its selective inhibition of acetylcholinesterase (AChE), but also as a dietary supplement in the USA [2,3,10,11]. Whole *H. serrata *plants are the original source of Hup A production. *P. carinatus *is commonly characterized as low, evergreen and coarsely moss-like, has a close taxonomic relationship with *H. serrata *and also produces Hup A across the entire plant [2]. Given their great health benefits and economic value, these plants are in danger of extinction in China due to extensive collection for Hup A production [2,3]. To protect the *Huperziaceae *from extinction and to support efforts to produce Hup A, cultivation and *in vitro *propagation have been investigated [12]. However, the long-term and efficient protection of these plants will depend on research into the molecular mechanisms of lycopodium alkaloid biosynthesis and further research is required in that field so that Hup A can be produced using biotechnological approaches.

Several recent studies identified a type III polyketide synthase (PKS) and its corresponding gene in *H. serrata *[13,14]. A proposed biosynthesis pathway for the synthesis of Hup A and related lycopodium alkaloids consisting of pelletierine coupled with 4PAA was reported by Ma and Gang (2004) [4] based on previous studies [15-18]. Initially, L-lysine is decarboxylated by lysine decarboxylase to form cadaverine. The subsequent reactions on the intermediates include a series of oxidations, decarboxylations and N-methylations that lead to the production of Hup A and related lycopodium alkaloids [4,19]. Cytochrome P450s are proposed to catalyze the oxidative modifications of the intermediates [4]. However, the complete biosynthetic pathways of these alkaloids (including Hup A) have yet to be elucidated. Genomic and transcriptomic knowledge is currently restricted to ten *H. serrata *and three *P. carinatus *nucleotide sequences available in GenBank (January 2009). The limited genetic information available on these plants and our desire to identify genes involved in Hup A biosynthesis led us to analyze the transcriptomes of *H. serrata *and *P. carinatus*. The goal of this study was to analyze and compare the transcriptomes of *H. serrata *and *P. carinatus *using high-throughput sequencing of expressed sequence tags (ESTs) to discover functional genes, especially those involved in the biosynthesis of bioactive compounds and transcriptional regulation.

EST analysis is a cost-effective and rapid tool for the isolation of genes. It provides a route for the identification of novel genes and it allows characterization of the transcriptome in various tissues [20-22]. Ohlrogge and Benning (2000) [23] reviewed the discovery of genes involved in the biosynthesis of secondary metabolites using EST analysis. EST sequencing has also been used to establish phylogenetic relationships and identify simple sequence repeat (SSR) analysis. EST-SSRs have revealed polymorphisms not only within the source taxon, but in related taxa as well [24]. Recently, a "next-generation" high-throughput sequencing method based on the Roche 454 Genome Sequencer (GS) FLX platform has emerged as a cost-effective approach that is well adapted to the analysis of the transcriptomes of both model and non-model species [25-29]. Not only can the 454 sequencing technology identify a large number of expressed sequences, but it can also discover new genes via deep sequencing, effectively revealing the expression of many rare transcripts.

This report describes a 454-EST analysis of *H. serrata *and *P. carinatus *and discovers a large number of candidate transcripts that have significant sequence similarities to cytochrome P450s, methyltransferases and dioxygenases, all of which might be involved in lycopodium alkaloid biosynthesis in the *Huperziaceae *plants. Other genes involved in serratane (triterpenoid) and flavonoid biosynthesis as well as hormone biosynthesis and signal transduction were also found. Furthermore, SSRs were detected among the *H. serrata *and *P. carinatus *454-EST datasets. This 454-EST analysis provides the first insight into the expressed genes of *H. serrata *and *P. carinatus*, the representative members of the *Huperziaceae *family. Potential genetic markers as well as sets of transcripts involved in secondary metabolite biosynthesis (particularly of lycopodium alkaloid biosynthesis) and transcriptional regulation were obtained.

## Results and Discussion

### 454-EST sequencing and assembly

We constructed two cDNA libraries from a pool of mRNA from the vegetative organs of *H. serrata *and *P. carinatus *using the SMART technology. The two libraries were sequenced on a 454-GS FLX Titanium platform, a one-quarter plate run for *H. serrata *and a one-eighth plate run for *P. carinatus*. After initial quality filtering with the default parameters, these runs yielded a total of 57 Mb from 140,930 high-quality (HQ) sequence reads for *H. serrata *and 30 Mb from 79,920 HQ sequence reads for *P. carinatus *(Table 1). An overview of the sequencing and assembly processes is shown in Table 1. After removal of sequences shorter than 50 bp, a total of 136,968 (97.2%) *H. serrata *and 78,282 (97.9%) *P. carinatus *HQ reads with average lengths of 405 ± 129 and 382 ± 122 bp, respectively, were used in the assembly (Table 1). Most of the reads were similar in length to the results from those studies [28,29] and longer than those used in the previous 454-EST studies [30-32].

**Table 1:** Summary of *H. serrata *and *P. carinatus *454-EST data

	*H. serrata*	*P. carinatus*
Total bases	57,028,559 bp	30,498,684 bp
No. of HQ reads	140,930	79,920
Average read length	405 ±129 bp	382 ± 122 bp
No. of contigs	14,085	9,120
Average contig length	608 ± 460 bp	532 ± 380 bp
No. of contigs larger than 500 bp	8,948 (63.5%)	3,020 (33.1%)
No. of singletons	22,678	22,692
Average singleton length	351 ± 165	353 ± 132
No. of singletons above 200 bp	16,879	18,822
No. of unique putative transcripts	36,763	31,812
Annotated sequences^a^	16,274 (44.3%)	14,070 (44.2%)

^a^The unique putative transcripts were annotated by BLASTX analysis against the NCBI nr database.

**Figure 1:**
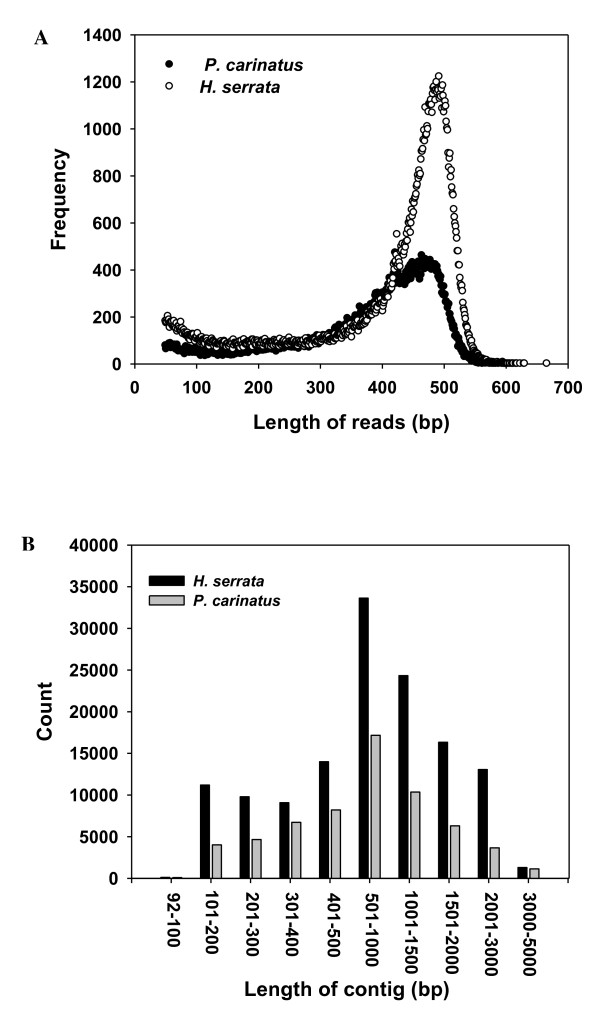
**Distribution of read lengths and contig lengths for *H. serrata *and *P. carinatus*.** A. Size distribution of 454 sequencing reads after removal of adaptor sequences. B. Length distribution of contigs (assembled sequences) in the 454-EST datasets.

The length distribution for these reads is given in Fig 1A, and it suggests that approximately 90% of the reads were between 200 and 600 bp in length. Clustering and assembly using the GS *De Novo *Assembler software v2.0.01 (454 Life Sciences, Roche) led to the construction of 14,085 and 9,120 contigs from *H. serrata *and *P. carinatus*, respectively, with average lengths of 608 ± 460 and 532 ± 380 bp. Among these, 8,948 (63.5%) *H. serrata *and 3,020 (33.1%) *P. carinatus *contigs with lengths longer than 500 bp were considered large contigs, the proportions of which were higher than the 8-27% reported in previous studies using 454 platforms [30,32]. The average depth of coverage for all *H. serrata *and *P. carinatus *contigs was 4.05-fold and 3.92-fold, respectively, which are lower than the coverage depths in previous studies [31,32]. The length distribution of the contigs from *H. serrata *and *P. carinatus*, summarized in Fig 1B, demonstrates that the majority of contigs had lengths of 500 to 1,500 bp. The relationship between the length of a given contig and the number of reads assembled into that contig was positive [32]. Overall, these results indicate the potential for high-throughput sequencing based on the 454-GS FLX Titanium to improve sequencing efficiency in future transcriptome analyses. There were 22,678 *H. serrata *and 22,692 *P. carinatus *sequences considered to be singletons, with average lengths of 351 ± 165 and 353 ± 132 bp, respectively (Table 1). In total, 36,763 *H. serrata *and 31,812 *P. carinatus *unique putative transcripts consisting of contigs and singletons were used for the annotation and subsequent analysis (Table 1).

### Annotation of unique putative transcripts

The annotation approach was based on sequence similarity searches in databases. To detect any possible contaminating ribosomal RNA sequences, all unique putative transcripts were subjected to a BLASTN search against the NCBI non-redundant nucleotide database, yielding 9,299 *H. serrata *and 7,910 *P. carinatus *well-identified sequences with E values ≤ 10^-5^. These included only 27 and 10 ribosomal RNA sequences from the *H. serrata *and *P. carinatus *454-EST datasets, respectively. To capture the most informative and complete annotations, all unique putative transcripts were first used in BLASTX searches against the SwissProt database. Approximately 20% of the unique putative transcripts (9,125 *H. serrata *and 7,555 *P. carinatus*) were thus annotated. Then, all the unique putative transcripts were subjected to a BLASTX search against the NCBI non-redundant protein database, yielding 16,274 and 14,070 well-identified sequences from *H. serrata *and *P. carinatus*, respectively, with at least one hit with an E value ≤ 10^-5 ^(Table 1). Altogether, only approximately 40% of the unique putative transcripts from *H. serrata *and *P. carinatus *showed significant sequence similarities to proteins in these databases, indicating that there is limited information about the genomes or transcriptomes of these ferns and their related species. In addition, this low proportion (about 40%) of unique putative transcripts assigned annotations might be due to the following reasons: (1) some singletons might be junk sequences produced by 454 sequencing, leading to assembly difficulties and errors, (2) the advances of deep sequencing, which can discover novel genes with low expression levels, (3) the shortness of the sequence reads, resulting in inefficient annotation.

Among the annotated unique putative transcripts, we found many transcripts that could be involved in the biosynthesis of lycopodium alkaloids and other types of bioactive compounds, including triterpenoids and flavonoids. These substantial 454-EST datasets provide comprehensive information about the *H. serrata *and *P. carinatus *transcriptomes for the first time.

### Comparison between *H. serrata *and *P. carinatus *based on GO and KEGG assignments

**Figure 2:**
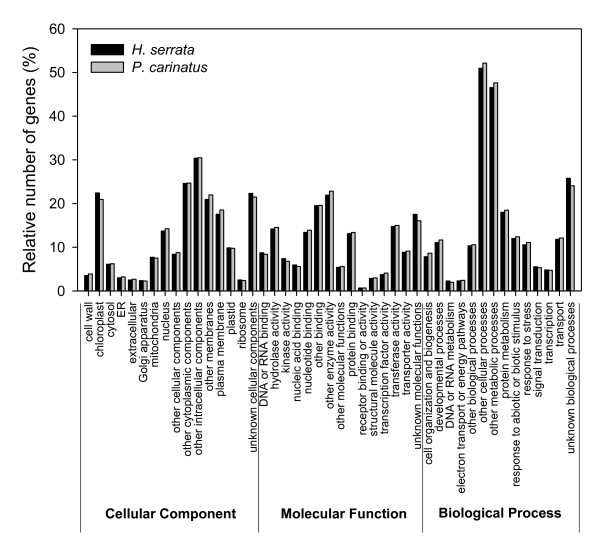
**Functional annotation of unique putative transcripts from *H. serrata *and *P. carinatus *based on GO categories**.

Gene Ontology (GO) assignments describe gene products in terms of their associated molecular functions, biological processes and cellular components. To assign putative functional roles to the unique putative transcripts from *H. serrata *and *P. carinatus*, we compared them to the *Arabidopsis *proteome using BLASTX and used the GO assignments of the *Arabidopsis *proteome, producing assignments for 14,770 (40.2%) *H. serrata *and 12,539 (39.4%) *P. carinatus *sequences, respectively, matching 8,117 and 7,061 *Arabidopsis *genes. The *H. serrata *and *P. carinatus *unique putative transcripts covered a similarly broad range of GO categories (Fig 2). The GO analysis identified well-represented categories within cellular components, including chloroplast, the plasma membrane and the nucleus. The sequences encoded a broad set of transcripts represented within the molecular function categories. The well-represented molecular function categories included transferase activity, hydrolase activity, nucleotide binding and transporter activity (Fig. 2). The functions of the identified genes cover various biological processes. Among these, other metabolic processes, protein metabolism, response to abiotic or biotic stimuli and transport were well-represented (Fig. 2). Three gene classes in biological processes (DNA or RNA metabolism, signal transduction and transcription) were overrepresented in the *H. serrata *library (Fig. 2). The other ten gene classes were slightly more abundant in the *P. carinatus *library (Fig. 2).

Kyoto Encyclopedia of Genes and Genomes (KEGG) analysis provides an alternative functional annotation of genes using Enzyme Commission (EC) numbers for assignments based on the genes associated with biochemical pathways. Overall, 13,174 *H. serrata *and 11,600 *P. carinatus *unique putative transcripts showed similarities to sequences in the KEGG databases. Of these sequences, only 3,189 for *H. serrata *and 2,752 for *P. carinatus *were assigned to biochemical pathways. The profiles of the genes from *H. serrata *and *P. carinatus *that were involved in biochemical pathways were similar (See Additional file 1). The KEGG metabolic pathways were well-represented by 1,123 *H. serrata *and 982 *P. carinatus *unique putative transcripts, most of which were related to amino acid metabolism, carbohydrate metabolism, energy metabolism, lipid metabolism and the biosynthesis of secondary metabolites (See Additional file 1). These findings probably result from high levels of expression of essential housekeeping genes, causing them to be well-represented in an incomplete transcriptome sequencing effort. In particular, the biosynthetic pathways of several bioactive compounds were well-represented by 115 *H. serrata *and 98 *P. carinatus *unique putative transcripts in our 454-EST datasets, totaling 41 *H. serrata*- and 44 *P. carinatus*-related EC numbers associated with the biosynthesis of alkaloids, flavones/flavonoids, phenylpropanoids, terpenoids and steroids (see Additional file 2). The KEGG pathways of genetic information processing, protein families and cellular processes were well-represented by unique putative transcripts from *H. serrata *and *P. carinatus *(see Additional file 1) . However, most of the unique putative transcripts (9,985 for *H. serrata *and 8,848 for *P. carinatus*) remained unassigned to any known biological pathway (See Additional file 1).

The distributions of gene functions and biochemical pathways (based on GO and KEGG assignments) in the *H. serrata *and *P. carinatus *transcriptomes were similar, particularly in the categories of molecular function and metabolism. These annotations provide valuable resources for investigations of gene function, cellular structures and processes in these two species. The discovery of transcripts that might be involved in the biosynthesis of alkaloids, flavones/flavonoids, phenylpropanoids and terpenoids will facilitate the elucidation of bioactive compound biosynthetic mechanisms at the molecular level in the *Huperziaceae *(see Additional file 2).

### SSR detection and validation

Microsatellites (or simple sequence repeats, SSRs) have been used as a resource for random candidate markers in population genetics studies. To better understand the natural diversity of *Huperziaceae *and to develop strategies for its sustainable utilization, we identified SSR motifs in our 454-EST dataset. Some of these EST-SSRs were tightly linked with functional genes that may control certain agronomic characters, including bioactive compound biosynthesis. A total of 2,729 and 1,573 putative SSR motifs from 2,308 (6.3%) and 1,343 (4.2%) SSR-containing unique putative transcripts were identified within the 454-ESTs of *H. serrata *and *P. carinatus*, respectively (see Additional file 3). The incidence of SSRs in the *H. serrata *(6.3%) and *P. carinatus *(4.2%) unique putative transcripts was lower than that in other plants such as apple (20%) [33], but it was similar to that of some dicotyledonous species (ranging from 2.65% to 16.82% [34]). The wide variation in the frequency of SSR motifs between different species results from the criteria used to identify SSRs during database mining [35] as well as from considerable variation in the abundance of SSRs across taxa [36]. Additionally, differences in the software used to detect SSRs might affect their frequency [37]. Among the SSRs, dinucleotides (1,766 for *H. serrata *and 1,214 for *P. carinatus*) were the most abundant repeat units followed by trinucleotides (722 for *H. serrata *and 299 for *P. carinatus*), tetranucleotides (117 for *H. serrata *and 29 for *P. carinatus*), hexanucleotides (65 for *H. serrata *and 20 for *P. carinatus*) and pentanucleotides (59 for *H. serrata *and 11 for *P. carinatus*). The occurrence of SSRs in *H. serrata *and *P. carinatus *is different from their distribution in other organisms such as the medicinal plant *E. sagittatum *[37] and cereal species [38], where trinucleotide repeat units are the most dominant SSR, followed by di- and tetranucleotide repeat units [37,38].

The relative frequency of repeats with different dinucleotide compositions was also biased towards one of four possible repeat classes in these two species (Table 2). Among the dinucleotide repeat classes, AG repeats were the most common dimer motif, followed by AC and AT. CG repeats were very infrequent in these two plants. Among the trinucleotide repeats, AGC/CAG/GCA/TGC/CTG/GCT was the largest repeat class followed by AAG/GAA/AGA/CTT/TTC/TCT (Table 2). The majority (81.8% for *H. serrata *and 82.8% for *P. carinatus*) of SSR-containing unique putative transcripts had a single SSR per sequence, while 421 (18.2%) *H. serrata *and 231 (17.2%) *P. carinatus *unique putative transcripts contained two or more SSRs per sequence. Over 50% (51.0% for *H. serrata *and 64.5% for *P. carinatus*) of the SSRs were between 9 and 14 bp long (see Additional file 3). These unique putative transcript-derived SSR markers that were generated in the present study provide a valuable genetic resource for future studies of these two species and other related species.

**Table 2:** Summary of di- and tri-nucleotide repeats in *H. serrata *and *P. carinatus *unique putative transcripts

Repeat composition	No. of unique putative transcripts (relative percentage)	
	***H. serrata***	***P. carinatus***
	
Dinucleotide		
AC/CA/GT/TG	301 (17.0%)	240 (19.8%)
AG/GA/CT/TC	1206 (68.3%)	805 (66.3%)
AT/TA	236 (13.4%)	135 (11.1%)
CG/GC	23 (1.3%)	34 (2.8%)
**Total**	**1766 (100%)**	**1214 (100%)**
Trinucleotide		
AAC/CAA/ACA/GTT/TTG/TGT	28 (3.9%)	35 (11.7%)
AAG/GAA/AGA/CTT/TTC/TCT	128 (17.7%)	58 (19.4%)
AAT/TAA/ATA/ATT/TTA/TAT	46 (6.4%)	19 (6.4%)
ACC/CAC/CCA/GGT/GTG/TGG	26 (3.6%)	8 (2.7%)
ACG/CGA/GAC/CGT/GTC/TCG	28 (3.9%)	10 (3.3%)
ACT/CTA/TAC/AGT/TAG/GTA	23 (3.2%)	10 (3.3%)
AGC/CAG/GCA/TGC/CTG/GCT	257 (35.6%)	105 (35.1%)
AGG/GGA/GAG/TCC/CTC/CCT	74 (10.2%)	22 (7.4%)
ATC/CAT/TCA/GAT/ATG/TGA	112 (15.5%)	31 (10.4%)
CCG/CGC/GCC/GGC/GCG/CGG	0	1 (0.3%)
**Total**	**722 (100%)**	**299 (100%)**

Twenty of the predicted SSR-containing sequences (ten singletons and ten contigs) from the *H. serrata *and *P. carinatus *454-ESTs were selected for validation using PCR and Sanger sequencing. A total of 16 sequences were amplified successfully including eight unique putative transcripts from *H. serrata *and eight from *P. carinatus *(see Additional file 4). Among these, nine contig sequences from *H. serrata *and *P. carinatus *were amplified by PCR, and all of these were found to contain SSR motifs. By comparison, only seven singleton sequences were amplified, five of which contained SSRs (see Additional file 4). Several singletons were not amplified successfully. The primers used to amplify these SSRs are listed in Additional file 4. Because the transcripts selected for validation include several candidate genes encoding enzymes involved in bioactive compound biosynthesis, the SSRs confirmed in these analyses may be valuable for investigating the genetic determinants of biosynthesis gene expression. In summary, the successful experimental validation of the majority of the computationally predicted SSR motifs confirms the utility of mining 454-ESTs for genetic markers. The development of EST-SSR markers in *Huperziaceae *should greatly facilitate marker-assisted selection, germplasm breeding, adulterated species identification and genetic diversity studies in these cultivated medicinal species.

### Genes related to lycopodium alkaloid biosynthesis

Both *H. serrata *and *P. carinatus *produce various types of lycopodium alkaloids, including Hup A. In an initial attempt to discover candidate genes involved in lycopodium alkaloid biosynthesis, we constructed a cDNA library from *H. serrata *leaves and used Sanger sequencing to generate 4,023 ESTs (HsESTs) [39]. Comparison of these HsESTs with the *H. serrata *454-ESTs showed that all of the HsESTs were included in the 454-EST dataset, reflecting the greater capacity of 454-ESTs for discovering genes.

Lycopodium alkaloids originate from the coupling of the pelletierine and the 4PAA/4PAACoA pathways [4,18] (see Additional file 5). In a first step, lysine decarboxylase decarboxylates L-lysine to form cadaverine. Thus, lysine decarboxylase is proposed to be the first enzyme that participates in lycopodium alkaloid biosynthesis [40]. Although investigations of this enzyme and its corresponding gene have not been performed in any *Huperziaceae *plant, we obtained a full-length cDNA sequence encoding this enzyme in a previous study [39]. Unique putative transcripts encoding lysine decarboxylase were also present in our 454-EST dataset (data not shown). The subsequent reactions from malonyl-CoA and Δ^1^- piperideine to pelletierine, the first general intermediate of lycopodium alkaloids, might be catalyzed by a diamine oxidase (enzyme B), a ketosynthase-type enzyme (enzyme C) or a decarboxylase (enzyme E) [4] (see Additional file 5A). The second general intermediate of all lycopodium alkaloids is phlegmarine, which is formed from pelletierine and 4PAA/4PAACoA, involving also a decarboxylation step [4,19] (see Additional file 5B). The oxidative modifications of phlegmarine and lycodane are presumably catalyzed by cytochrome P450 or dioxygenase, and they produce a series of precursors of Hup A [4] (see Additional file 5). Unique putative transcripts showing similarities to those of uncharacterized enzymes possibly associated with lycopodium alkaloid biosynthesis, including cytochrome P450-dependent monooxygenases (CYP450s), methyltransferases and dioxygenases, were found in the *H. serrata *and *P. carinatus *454-EST datasets (see Additional file 6, 7 and 8). These candidate CYP450s presumably catalyze the oxidative modifications leading to the production of lycopodium alkaloids (see Additional file 5B). Of the unique putative transcripts, 96 *H. serrata *and 82 *P. carinatus *sequences were annotated as putative CYP450s belonging to various subfamilies (see Additional file 6 and Additional file 9).

In plants, CYP450s catalyze many different reactions involved in a wide range of biosynthetic pathways, including those leading to alkaloids, phenylpropanoids, hormones, terpenoids, sterols, lignins, fatty acids, pigments and the detoxification of herbicides [41]. Although the results of several studies suggest progress in the isolation and characterization of CYP450s, identification of the range of functions of individual CYP450 proteins, which belong to diverse gene families and possess a variety of conserved domains, is a significant challenge for future research [42]. However, the identification of CYP72A1 from *Catharanthus roseus *[43] and CYP88D6 (licorice β-amyrin 11-oxidase) from *Glycyrrhiza uralensis *[44] provide examples of efficient approaches and the problems that may be encountered in the determination of biochemical functions of CYP450s.

**Figure 3:**
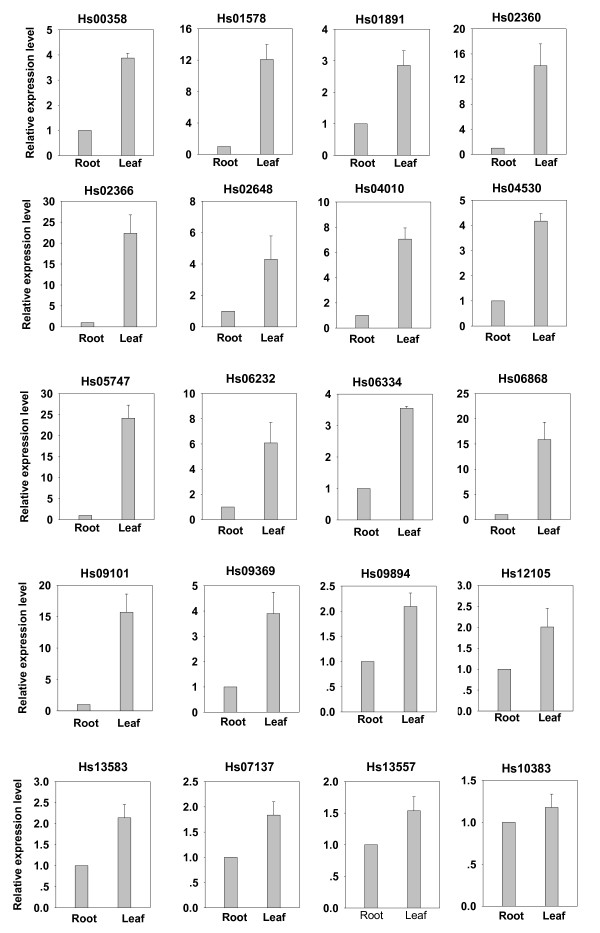
**Profile-based selection of candidate CYP450s most likely to be involved in lycopodium alkaloid biosynthesis from *H. serrata *using real-time PCR analysis**. Real-time PCR analysis of the transcripts with higher expression levels in *H. serrata *leaves than in roots.

Since lycopodium alkaloids are produced in both of *H. serrata *and *P. carinatus*, the relevant lycopodium CYP450s should be present in each of these two species. Therefore, to narrow down the list of likely candidate lycopodium CYP450 transcripts from the putative CYP450s, unique putative transcripts encoding CYP450s present in both species and showing sequence similarity were considered. These include a total of 63 unique putative transcripts (46 contigs and 17 singletons for *H. serrata *and 44 contigs and 19 singletons for *P. carinatus*) encoding putative CYP450s (see Additional file 10). The 46 contigs of *H. serrata *were selected for the subsequent real-time PCR analysis. Furthermore, due to the fact that the highest level of Hup A in *H. serrata *is in the leaves and the lowest level is in the roots [2], the genes involved in lycopodium alkaloid biosynthesis are expected to be expressed more abundantly in leaves than in roots. To assess changes in gene expression patterns on a functional level, the data were filtered by calculating the fold-change between the expression levels in different organs, and a comparison was made between leaves and roots. Thus, twenty putative CYP450s that are more abundantly expressed in *H. serrata *leaves than in roots were evaluated using real-time PCR analysis as likely candidate CYP450s involved in lycopodium alkaloid biosynthesis (Fig 3). Among the twenty putative *CYP450s*, The expression levels of Hs01578, Hs02360, Hs02366, Hs05747, Hs06868, and Hs09101 were significantly higher in leaves than in roots (> 10 times) as showed in Fig 3. The selection method by real-time PCR analysis of the candidate CYP450s used in this study was similar to that used for the identification of CYP88D6 in *G. uralensis *[44]. CYP88D6 was identified after mining of EST datasets and subsequent transcript profiling-based selection. The expression profile of *CYP88D6 *was consistent with the organ-specific accumulation pattern of glycyrrhizin [44]. The other 26 contigs detected *H. serrata *transcripts that were expressed more highly in roots than in leaves were not considered in our subsequent studies (data not shown).

**Figure 4:**
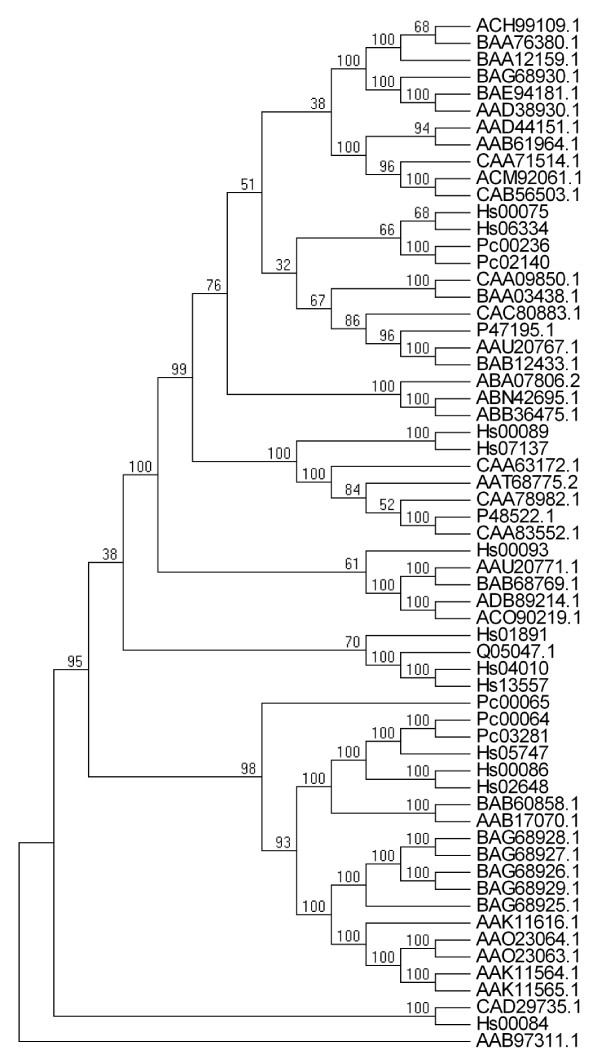
**Phylogenetic analysis of full-length CYP450 genes from *H. serrata *and *P. carinatus *and characterized CYP450 genes from other plants**. Amino acid sequences were aligned using the CLUSTALW program, and evolutionary distances were computed using MEGA4 with the Poisson correction method. Bootstrap values obtained after 1,000 replications are indicated on the branches. The GenBank/EMBL/DDBJ accession numbers of the sequences are: ACH99109.1 (*Camellia sinensis*), BAA76380.1 (*Glycyrrhiza echinata*), BAA12159.1 (*Glycine max*), BAG68930.1 (*Glycyrrhiza uralensis*), BAE94181.1 (*Glycine max*), AAD38930.1 (*Glycine max*), AAD44151.1 (*Mentha × piperita*), AAB61964.1 (*Solanum chacoense*), CAA71514.1 (*Glycine max*), ACM92061.1 (*Catharanthus roseus*), CAB56503.1 (*Catharanthus roseus*), CAA09850.1 (*Catharanthus roseus*), BAA03438.1 (*Petunia × hybrida*), CAC80883.1 (*Catharanthus roseus*), P47195.1 (*Berberis stolonifera*), AAU20767.1 (*Thalictrum flavum subsp. glaucum*), BAB12433.1 (*Coptis japonica*), ABA07806.2 (*Nicotiana tabacum*), ABN42695.1 (*Nicotiana tabacum*), ABB36475.1 (*Nicotiana tabacum*), CAA63172.1 (*Glycine max*), AAT68775.2 (*Camellia sinensis*), CAA78982.1 (*Helianthus tuberosus*), P48522.1 (*Catharanthus roseus*), CAA83552.1 (*Catharanthus roseus*), AAU20771.1 (*Thalictrum flavum subsp. glaucum*), BAB68769.1 (*Coptis japonica*), ADB89214.1 (*Papaver somniferum*), ACO90219.1 (*Eschscholzia californica*), Q05047.1 (*Catharanthus roseus*), BAB60858.1 (*Arabidopsis thaliana*), AAB17070.1 (*Solanum lycopersicum*), BAG68928.1 (*Lotus japonicus*), BAG68927.1 (*Lotus japonicus*), BAG68926.1 (*Medicago truncatula*), BAG68929.1 (*Glycyrrhiza uralensis*), BAG68925.1 (*Medicago truncatula*), AAK11616.1 (*Hordeum vulgare subsp. vulgare*), AAO23064.1 (*Pisum sativum*), AAO23063.1 (*Pisum sativum*), AAK11564.1 (*Arabidopsis thaliana*), AAK11565.1 (*Arabidopsis thaliana*), CAD29735.1 (*Solanum tuberosum*), AAB97311.1 (*Catharanthus roseus*). Hs: *H. serrata*; Pc: *P. carinatus*. The contigs of *H. serrata *and *P. carinatus *were represented by Hs or Pc plused five digits.

Besides the above selection, the other potential candidate CYP450 selected method using a phylogenetic analysis between the full-length CYP450s from our 454-EST datasets and other CYP450s from other plants which were associated with secondary metabolism biosynthesis was performed. More putative CYP450 transcripts were amplified and obtained the full-length cDNA sequences using RT-PCR and/or the RACE approach based on the contig sequences including the unique putative transcripts which were showed in Fig 3. However, the amplifications of the unique putative transcripts presented in Fig 3 such as Hs02360, Hs02366, Hs06232, Hs06868, Hs09101, Hs09894 and Hs12105 from *H. serrata *using the RACE approach were unsuccessful. Together, a total of seventeen full-length CYP450s from our 454-EST datasets (including twelve genes from *H. serrata *and five genes from *P. carinatus*) were used in phylogenetic analysis. The phylogenetic relationship between these CYP450s and characterized CYP450s from other plants is depicted in Fig 4. Three CYP450s (Hs01891, Hs04010, and Hs13557) belong to a group with CYP450 (Q05047.1) encoding secologanin synthase (SLS), which catalyzes the oxidative cleavage of loganin into secologanin [45] (Fig. 4). These candidate CYP450s were also expressed more abundantly in leaves than in roots (Fig. 3), which is consistent with the organ-specific accumulation pattern of lycopodium alkaloids and thus they may play key roles in alkaloid biosynthesis. It is also noteworthy that the CYP450 of Hs00093 is phylogenetically close to these CYP450s (AAU20771.1, BAB68769.1, ADB89214.1 and ACO90219.1) (Fig. 4). AAU20771.1 encoding (S)-canadine synthase catalyzes the formation of the methylenedioxy bridge in berberine synthesis [46]. BAB68769.1 encodes a methylenedioxy bridge-forming cytochrome P450 dependent enzyme, while ADB89214.1 and ACO90219.1 encode stylopine synthase, all of which are involved in (S)-stylopine biosynthesis [47]. Phlegmarine might be modified by an enzyme similar to the berberine bridge enzyme to form lycodane [4]. Therefore, Hs00093 is a lead candidate for involvement in lycopodium alkaloid biosynthesis. In subsequent studies, we will clone the full-length cDNA sequences for these candidates and assess their enzymatic activities in lycopodium alkaloid biosynthesis. In addition, according to the phylogenetic analysis, the CYP450s from one group (including the four genes Hs00075, Hs06334, Pc00236 and Pc02140) might be involved in flavone/flavonoid biosynthesis, and those in another group (including the six genes Pc00064, Pc03281, Pc00065, Hs05747, Hs00086 and Hs02648) might be involved in terpenoid biosynthesis (Fig. 4).

The CYP450 enzymes involved in lycopodium alkaloid biosynthesis are neither well-characterized nor well-understood. Thus, these candidate CYP450s generated in our 454-EST datasets will be a valuable resource for CYP450 identification in *H. serrata *and *P. carinatus*. In addition, unique putative transcripts that show similarities to dioxygenases and methyltransferases are presented in Additional files 7 and 8, and they will be screened using the transcript profiling-based selection method described above for selecting CYP450s. These genes will be further characterized for their biological functions in the biosynthesis of lycopodium alkaloids, which will allow for identification of specific enzymes and elucidation of the entire biosynthetic pathway(s) of the lycopodium alkaloids based on the approaches of modern biochemistry and molecular biology.

### Genes related to terpenoid and flavone/flavonoid biosynthesis

The serratanes, a unique family of pentacyclic triterpenoids possessing seven tertiary methyls and a central seven-member C-ring, were isolated from plants of the *Pinaceae *and *Lycopodium *species [48]. Previous studies have revealed the presence of serratane-type triterpenoids in the medicinal plants of *H. serrata *[49-51]. The mevalonate pathway (MVA pathway) and methylerythritol phosphate pathway (MEP pathway) play important roles in the biosynthesis of terpenoids by providing precursors for these processes. Sequences representing enzymes involved in terpenoid biosynthesis, including most of the steps of the MEP and MVA pathways, were abundant in our 454-EST dataset (see Additional file 2). Unique putative transcripts with homology to known key enzymes in steroid biosynthesis were found in these ESTs, including transcripts for squalene synthetase, farnesyl pyrophosphate synthetase, geranylgeranyltransferase, isopentenyl-diphosphate Δ-isomerase and squalene monooxygenase (see Additional file 2).

Flavonoids, including anthocyanins and flavanols, are polyphenolic secondary metabolites derived from the amino acid phenylalanine and synthesized via the phenylpropanoid pathway [52]. These secondary metabolites possess important beneficial health attributes, probably due to their antioxidant activity [53,54]. The identification of genes specific to the flavonoid biosynthetic pathway and the isolation of flavone glycosides from *H. serrata *suggest that flavonoids, including anthocyanins and flavones, are constituents of this medicinal plant [14,55]. Representative genes involved in flavonoid biosynthesis were found by BLASTX searches for unique putative transcripts representing different enzymes in the pathway. There were multiple unique putative transcripts for genes in this pathway, including transcripts encoding cinnamate 4-hydroxylase, chalcone synthase, chalcone isomerase, NAD(P)H-dependent 6'-deoxychalcone synthase and flavonoid 3',5'-hydroxylase (F3'5'H) (see Additional file 2). In the pathway for the production of anthocyanins, unique putative transcripts encoding leucoanthocyanidin dioxygenase, anthocyanidin synthase and anthocyanidin 3-O-glucosyltransferase were also prominently found in the *H. serrata *and *P. carinatus *454-EST datasets (see Additional file 2).

### Genes related to growth and development

The *Huperziaceae *are some of the oldest vascular plants, and they grow very slowly, usually in special habitats. Some classes of phytohormones (e.g., gibberellins and abscisic acid) and transcription factors might play key roles in the regulation of plant development and environmental responses in the *Huperziaceae *plants.

Phytohormones, such as auxin, gibberellins (GAs), abscisic acid (ABA), cytokinins, ethylene and brassinosteroids (BRs), play critical roles in the regulation of diverse aspects of plant growth and development as well as in environmental responses [56-58]. The biosynthetic pathways of hormones have been elucidated in several model organisms [59-61]. The 454-EST database from *H. serrata *and *P. carinatus *contains sequences with homology to many known enzymes involved in the biosynthesis of phytohormones (see Additional file 11).

Unique putative transcripts showing sequence similarities to gibberellin 2-beta-dioxygenase, gibberellin 3-beta-dioxygenase and gibberellin 20-oxidase, all of which are involved in GA biosynthesis, were found in the 454-EST datasets from *H. serrata *and *P. carinatus *(see Additional file 11). There were multiple sequences for genes encoding abscisic acid 8'-hydroxylase in the catabolic pathway of ABA from *H. serrata *and *P. carinatus *(see Additional file 11). In addition, unique putative transcripts with homology to 1-aminocyclopropane-1-carboxylate oxidase, 1-aminocyclopropane-1-carboxylate synthase and 1-aminocyclopropane-1-carboxylate deaminase, which are involved in the biosynthesis of ethylene, were abundant in the *H. serrata *and *P. carinatus *454-EST datasets. Additionally, unique putative transcripts with homology to cytokinin-O-glucosyltransferase and cytokinin dehydrogenase, which are involved in the biosynthesis of cytokinin, were also abundant in the *H. serrata *and *P. carinatus *454-EST datasets (see Additional file 11). Some components involved in hormone signal transduction were also discovered, such as the gibberellin receptors GID1L1, GID1L2, GID1L3 and GID1; the ethylene receptor; the auxin response factor; and brassinosteroid insensitive 1-associated receptor kinase 1 (See Additional file 11). These genes might have essential functions in the regulation of development and/or environmental responses in *H. serrata *and *P. carinatus*.

Transcription factors play key roles in the regulation of gene expression in response to developmental processes and environmental stress in plants [62]. In this study, we detected 504 *H. serrata *and 469 *P. carinatus *unique putative transcripts representing homologs belonging to different transcription factor (TF) families, including the MYB, homeobox, zinc finger, basic helix-loop-helix, bZIP, WRKY, AUX/IAA, ARF and B3 family proteins (see Additional file 12). The most abundant TF family in our datasets was the MYB protein family, which is characterized by conserved domains. Another highly expressed set of transcription factors was the homeobox family in our datasets. Interestingly, unique putative transcripts encoding B3 domain transcription factors were expressed abundantly in *P. carinatus *(26 unique putative transcripts) but were absent in the *H. serrata *454-EST dataset (see Additional file 12). This was a surprising observation, considering the fact that the two species are so closely related. It may be that the timing of material collection corresponded to a period of inactivity of these genes, which would explain the lack of the corresponding transcripts in the *H. serrata *454-EST dataset.

We also searched for transcription factor genes that are expressed in spatial and temporal patterns indicative of important roles in the regulation of secondary metabolite biosynthesis. Several transcription factors involved in the regulation of terpenoid indole alkaloid biosynthesis genes have been isolated and studied [63]. Some transcription factors serve as positive or negative regulators in the biosynthesis of secondary metabolites. The molecular functions of the transcription factors which were expressed abundantly in *H. serrata *and *P. carinatus *(see Additional file 12) potentially involved in biosynthesis of secondary metabolites and/or environmental responses will be characterized in a future study.

## Conclusions

This collection of 454-ESTs from *H. serrata *and *P. carinatus *provides a significant resource for gene discovery in these medicinal plants. The results outlined here establish a rapid and cost-effective method for deep transcriptome sequencing using the 454-GS FLX Titanium platform. The 454-EST datasets from *H. serrata *and *P. carinatus *provide a description of the expressed genes in these two species, which belong to different genera but the same family. In addition to finding a large number of genetic markers for studies of genetic connectivity and structure, we discovered candidate genes that provide the foundation for functional research on secondary metabolism and growth regulation in the *Huperziaceae*. These unique putative transcripts are likely candidates for the biosynthesis of lycopodium alkaloids, terpenoids and flavones/flavonoids, as well as associated with developmental regulation and environmental responses. Our initial *H. serrata *and *P. carinatus *454-EST analysis represents a substantial contribution that will increase the opportunities for specific gene discovery and pathway-based studies.

## Methods

### Plant material

*H. serrata *and *P. carinatus *plants (grown in the wild) that had reached heights of 10-12 and 50-55 cm, respectively, were collected in Bawangling at an altitude of 1,320 meters (109°10′ E, 19°7′ N) in Hainan Province (November 3, 2008). The plants were authenticated by Professor Yu-Lin Lin of the Institute of Medicinal Plant Development (IMPLAD) using the morphological identification approach of the Flora of China and the Flora of Hainan Province, Chinese Academy of Medical Sciences. Whole plants were collected and rinsed with water 5-8 times. Then, the plants were dried gently and quickly with absorbent paper. The cleaned leaves, stems and roots were isolated and frozen in liquid nitrogen immediately, then stored at -70°C until RNA isolation.

### RNA preparation

Total RNA was isolated from six grams (two grams each of roots, stems and leaves) of the whole plant using the RNeasy plant kit (BioTeke, Beijing, China). The RNA quality was tested using EtBr-stained agarose gels, and the concentration was assessed using a GeneQuant100 spectrophotometer (GE Healthcare, UK). Approximately two micrograms of poly(A) RNA was isolated from total RNA using an Oligotex^® ^mRNA Midi Kit (QIAGEN, CA), and the quality was assessed with a GeneQuant100 spectrophotometer (GE Healthcare, UK) prior to cDNA synthesis. The cDNA was produced using 0.8 micrograms of purified poly(A) RNA according to the manufacturer's instructions provided with Clontech's SMART cDNA synthesis kit (Clontech, USA). For the two libraries, cDNA was amplified using PCR Advantage II polymerase (Clontech, USA) and the following thermal profile: 1 min at 95°C followed by 13 cycles of 95°C for 15 sec, 65°C for 30 sec, and 68°C for 6 minutes. Five microliters of PCR product were electrophoresed in a 1% agarose gel to determine amplification efficiency. Approximately 3-5 micrograms of amplified cDNA PCR product were purified using the PureLink™ PCR purification kit (Invitrogen, USA) before the 454 library construction.

### Library construction and 454 sequencing

The two 454 libraries were constructed as described previously [64]. Approximately five micrograms of amplified cDNA was sheared by nebulization to produce random fragments approximately 300-800 bp in length for 454-sequencing. The fragmented cDNA samples were assessed by gel electrophoresis to evaluate the effectiveness of the process. Oligonucleotide adaptors were ligated to the fragmented cDNA samples according to standard procedures. The DNA fragments were then denatured to generate single-stranded DNA that was amplified by emulsion PCR for sequencing. The sequencing of the libraries was performed on a 454-GS FLX Titanium sequencing platform (454 Life Sciences, Roche). All raw 454 sequence data generated in this study are available in the Sequence Read Archive of the National Center for Biotechnology Information (NCBI) [65] with the accession numbers SRX010999 for *H. serrata *and SRX011001 for *P. carinatus*.

### Sequence assembly

All analyses of the sequencing data were performed with the GS FLX Software v2.0.01 (454 Life Sciences, Roche). After using a series of normalization, correction and quality-filtering algorithms, the 454 data were filtered for weak signals and low-quality sequences, and the read ends were screened and trimmed for 454 adaptor sequences to yield high quality (HQ) sequences (> 99.5% accuracy on single base reads). A subsequent filtering step included the masking of SMART PCR primer sequences (Clontech) and the removal of sequences shorter than 50 bp before assembly. Finally, these HQ reads were assembled into unique putative transcripts (including contigs and singletons) using GS *De Novo *Assembler Software, which is an application of the GS FLX Software. The assembly was performed using the default parameters.

### Sequence annotation

To find the most descriptive annotation possible for each sequence, sequence annotation was based on a set of BLAST searches. The BLAST software version was BLAST 2.2.17 (downloaded from NCBI). The sequences were searched using BLASTN against the NCBI non-redundant nucleotide (nt) database with an E-value cut-off of 10^-5^. The first BLASTX search was used to search for similar sequences in the SwissProt database (http://www.uniprot.org/, released on 06/19/2009), then the NCBI non-redundant protein (nr) database (released on 06/23/2009) was searched. The top hit of each BLASTX search with an E-value ≤ 1 × 10^-5 ^was considered a significant match. The functional categories of these unique putative transcripts were further identified using the Gene Ontology (GO) Database. The unique putative transcripts were categorized according to GO on the basis of AGI codes and The Arabidopsis Information Resource (TAIR) GO slim provided by TAIR. The *Arabidopsis *proteome data were downloaded from TAIR http://www.arabidopsis.org (version Tair9). The biochemical pathway assignments were carried out according to Kyoto Encyclopedia of Genes and Genomes (KEGG) mapping http://www.genome.ad.jp/kegg/kegg2.html (version KEGG 50). Enzyme Commission (EC) numbers were assigned to the unique putative transcripts based on BLASTX with a cut-off value of *E *≤ 10^-5 ^upon searching protein databases. The sequences were mapped to KEGG biochemical pathways according to the EC distribution in the pathway databases.

### SSR detection and validation

The detection of SSRs in the total unique putative transcripts from *H. serrata *and *P. carinatus *was performed using the Simple Sequence Repeat Identification Tool (SSRIT) http://www.gramene.org/db/markers/ssrtool, which accepts FASTA-formatted sequence files and reports the sequence ID, the SSR motif, the number of repeats (di-, tri-, tetra-, penta- or hexa-nucleotide repeat units), the repeat length, the position of the SSR and the total length of the sequence in which the SSR was found [66]. Only repeat lengths longer than 9 bp were used in the analysis. The repeat classes (e.g., di-, tri-, tetra-, penta- or hexa-nucleotides) were combined by type; for example, GA repeats also encompassed repeats identified as AG and their complementary sequences TC and CT repeats. For trinucleotides, AAC repeats include CAA and ACA, as well as their complementary sequences GTT, TTG and TGT. The search parameter for the maximum motif-length group was set to hexamer and that for the minimum number of repeats was set to five.

SSR validation was performed using RT-PCR and the Sanger sequencing method. According to the SSR positions in the unique putative transcripts, we selected sequences on the left and right of an SSR motif (with the distance between the primers and the SSR motif at least 100 bp) to design specific primers. The parameters for designing the primers were as follows: primer length ranging from 18 bases to 25 bases with 20 as the optimum, PCR product size ranging from 100 to 500 bp, optimum annealing temperature 58°C, and GC content from 40% to 60% with 50% as the optimum. Total RNA was extracted using the RNeasy plant kit (BioTeke, Beijing, China) from whole plants similar to the plant material used in the high throughput sequencing. Reverse transcription reactions were performed using 3 μg of total RNA and a SuperScript III RT kit (Invitrogen) in a 20 μl volume at 50°C for 50 min. Using 10 ng of the amplified intact cDNA as a template, each target was amplified separately in a 25 μl volume containing 1 × PCR buffer, 0.2 μM each specific primer, 0.4 mM dNTP and 0.5 U Pyrobest™ DNA polymerase (Takara, Dalian, China). The amplification profile consisted of an initial denaturation step at 94°C for 4 min, followed by 35 cycles of 94°C for 30 s, 58°C for 30 s and 72°C for 40 s, with a final extension step at 72°C for 10 min. After evaluating their molecular weight and specificity using gel electrophoresis, the PCR products were divided into two groups: one group with bright specific bands was used for sequencing using the specific forward primers, and the others were ligated into the vector pMD18-T (Takara), transformed into Top 10 *E. coli *competent cells (Takara) and sequenced using RV-M primers.

### Real-time PCR analysis

To assay the transcript levels of putative CYP450s in roots and leaves of *H. serrata*, quantitative real-time PCR analysis was performed with an IQ5 Multicolor Real-Time PCR Detection System (BIO-RAD, USA) using Power SYBR^R ^Green PCR Master Mix (Applied Biosystems, Warrington, UK), and it was repeated three times. Total RNA was extracted from *H. serrata *roots and leaves and treated with RNase-free DNase (TaKaRa). The reverse transcription reaction was carried out using Oligod(T)_15 _primers and SuperScript III (RT kit; Invitrogen) following the manufacturer's recommendations. Each reaction contained 10 μl 2 × SYBR Green Master Mix Reagent (Applied Biosystems), 10 ng of cDNA sample and 200 nM gene-specific primers. The total volume was 20 μl. The cycling conditions were: 50°C for 2 min and 95°C for 5 min, followed by 40 cycles of 95°C for 15 sec and then 60°C for 1 min. This program was followed by a melting-curve program, 55 to 85°C, with a 5 s hold at each temperature. The mean value of three replicates was normalized using *Actin *(Hs00917 in our *H. serrata *454-EST dataset) as the internal control. PCR amplification was performed using specific primers for the putative *CYP450s *listed in Additional file 4. The suitability of the oligonucleotide sequences in terms of annealing efficiency was evaluated in advance using the Primer 3.0 program. The mean value of three replicates was normalized using *Actin *as the internal control. The relative expression levels were calculated by comparing the CTs (cycle thresholds) of the target genes with that of the housekeeping gene *Actin *using the 2^-ΔΔCT ^method [67].

### Phylogenetic analysis

Distances between the clones were calculated with the CLUSTAL W program [68]. The scale represents 0.1 amino acid substitutions per site. Amino acid sequences were aligned using the CLUSTALW program and evolutionary distances were computed using the Poisson correction method, and a neighbor-joining (NJ) tree was constructed with MEGA4. Bootstrap values obtained after 1,000 replications, which are given on the branches.

## List of abbreviations

ABA: abscisic acid; BLAST: Basic Local Alignment Search Tool; bp: base pair; BR: brassinosteroid; cDNA: complementary DNA; EST: expressed sequence tag; GA: gibberellin; GGDP: geranylgeranyl diphosphate; GO: Gene Ontology; Huperzine A: Hup A; KEGG: Kyoto Encyclopedia of Genes and Genomes; NCBI: National Center for Biotechnology Information; SLS: secologanin synthase; SSRs: simple sequence repeats; TF: transcription factor.

## Authors' contributions

HML participated in the design of the study, contributed to the tissue sample collection, RNA extraction and 454-library construction, analyzed the data and drafted the manuscript. YL contributed to the bioinformatic analysis and helped with the construction of the 454-libraries. CS participated in the study design and discussed the results. QW helped to analyze the SSRs. JYS initiated the EST projects and discussed the results. YZS helped analyze the real-time PCR results and with the phylogenetic analysis. AS contributed to the discussion of secondary metabolite biosynthetic gene candidates, especially regarding the selection of the likely lycopodium CYP450 candidates, and helped revise the manuscript. This work was conducted in the laboratory of SLC, who initiated the 454-sequencing projects and contributed to the evaluation and discussion of the results, as well as contributed to the revision of the manuscript. All authors read and approved the final manuscript.

## Supplementary Material

Additional file 1 **Mapping of *H. serrata *and *P. carinatus *unique putative transcripts to KEGG biochemical pathways**. List of the numbers of *H. serrata *and *P. carinatus *unique putative transcripts involved in metabolism, genetic information processing, environmental information processing, cellular processes, protein families, human diseases and unclassified in the 454-EST datasets. Click here for file

Additional file 2 **Key enzyme discovery**. Unique putative transcripts encoding key enzymes involved in the biosynthesis of alkaloids, flavones/flavonoids, phenylpropanoids, terpenoids and steroids from *H. serrata *(Sheet 1) and *P. carinatus *(Sheet 2). Enzyme names are listed together with their corresponding EC number and their unique putative transcript ID. Click here for file

Additional file 3 **SSR discovery**. List of unique putative transcripts (including contigs and singletons) containing microsatellite loci from the *H. serrata *(Sheet 1) and *P. carinatus *(Sheet 2) 454-EST datasets, including the unique putative transcript ID as well as the type (motif), number and length of the SSR repeat. Click here for file

Additional file 4 **Validation of the SSR-containing unique putative transcripts (including contigs and singletons) by PCR amplification and Sanger sequencing**. Unique putative transcripts chosen from the *H. serrata *and *P. carinatus *454-EST dataset, including five singletons and five contigs for each species. Successful amplifications of these sequences and detections of SSRs are marked with "+" or "√", respectively; unsuccessful amplifications and detections are marked as "-". The primers used to amplify the sequences are also listed. The contigs were represented by Hs or Pc plused five digits and the singletons were represented by Pc plused 14 letters. Click here for file

Additional file 5 **Proposed biosynthetic pathways for Hup A and related lycopodium alkaloids in *Huperziaceae *plants**. Proposed biosynthetic pathways for Hup A and related lycopodium alkaloids (From Ma and Gang, 2004). **A**. Proposed biosynthetic pathways for the precursors pelletierine and 4PAA. **B**. Proposed biosynthetic pathways from pelletierine and 4PAA to Hup A and related lycopodium alkaloids. Click here for file

Additional file 6 **Cytochrome P450 discovery**. Unique putative transcripts from *H. serrata *(Sheet 1) and *P. carinatus *(Sheet 2) with sequence similarities to cytochrome P450s. Click here for file

Additional file 7 **Methyltransferase discovery**. Unique putative transcripts from *H. serrata *(Sheet 1) and *P. carinatus *(Sheet 2) with sequence similarities to methyltransferases. Click here for file

Additional file 8 **Dioxygenase discovery**. Unique putative transcripts from *H. serrata *(Sheet 1) and *P. carinatus *(Sheet 2) with sequence similarities to dioxygenases. Click here for file

Additional file 9 **Summary of the CYP subfamily in the *H. serrata *and *P. carinatus *454-EST database**. The number of unique putative transcripts encoding putative CYP450s from *H. serrata *and *P. carinatus *belonging to different subfamilies. Click here for file

Additional file 10 **Unique putative transcripts encoding putative CYP450s with sequence similarity between *H. serrata *and *P. Carinatus***. List of *H. serrata *contigs and singletons encoding putative CYP450s showing sequence similarity to *Ph. carinatus *unique putative transcripts. Click here for file

Additional file 11 **Transcripts related to phytohormones**. Unique putative transcripts from *H. serrata *(Sheet 1) and *P. carinatus *(Sheet 2) with similarities to genes involved in the biosynthetic, catalytic, or signal transduction processes of phytohormones. Click here for file

Additional file 12 **Major transcription factor families identified from *H. serrata *and *P. carinatus *using Inter-Pro**. Unique putative transcripts from *H. serrata *(Sheet 1) and *P. carinatus *(Sheet 2) with similarities to genes encoding transcription factors. Click here for file
