# Supplementary material for: Comparison of 454-ESTs from Huperzia serrata and Phlegmariurus carinatus reveals putative genes involved in lycopodium alkaloid biosynthesis and developmental regulation
Source: BMC Plant Biol. 2010 Sep 21;10:209. doi: 10.1186/1471-2229-10-209 (PMC2956558; doi:10.1186/1471-2229-10-209)
Supplement: Additional file 1 — Mapping of H. serrata and P. carinatus unique putative transcripts to KEGG biochemical pathways. List of the numbers of H. serrata and P. carinatus unique putative transcripts involved in metabolism, genetic information processing, environmental information processing, cellular processes, protein families, human diseases and unclassified in the 454-EST datasets. [file 1471-2229-10-209-S1.DOC]

## Table S1: Mapping of *H*. *serrata* and *P. carinatus* unique putative transcripts to KEGG biochemical pathways

| **KEGG categories represented** | **No. of unique putative transcripts** | |
| --- | --- | --- |
|  | ***H*. *serrata*** | ***P. carinatus*** |
| **Metabolism 1,123 982** | | |
| Amino Acid Metabolism | 302 | 283 |
| Biosynthesis of Secondary Metabolites | 96 | 64 |
| Carbohydrate Metabolism | 266 | 243 |
| Energy Metabolism | 124 | 110 |
| Glycan Biosynthesis and Metabolism | 60 | 38 |
| Lipid Metabolism | 109 | 108 |
| Other | 166 | 136 |
| **Genetic Information Processing 661 557** | | |
| Folding, Sorting and Degradation | 215 | 214 |
| Replication and Repair | 103 | 58 |
| Transcription | 67 | 51 |
| Translation | 276 | 234 |
| **Environmental Information Processing 139 131** | | |
| Membrane Transport | 68 | 51 |
| Signal Transduction | 69 | 78 |
| Signaling Molecules and Interaction | 2 | 2 |
| **Cellular Processes 292 265** | | |
| Cell Communication | 60 | 52 |
| Cell Growth and Death | 95 | 87 |
| Cell Motility | 21 | 17 |
| Other | 116 | 109 |
| **Protein Families 583 489** | | |
| **Human Diseases 181 144** | | |
| **Unclassified 210 183** | | |
| **Unassigned^a^** | **9,985** | **8,848** |

^a^Unassigned unique putative transcripts are those that have significant similarities to known sequences in the KEGG database that have undefined involvement biochemical pathways.
