# Supplementary material for: Comparison of 454-ESTs from Huperzia serrata and Phlegmariurus carinatus reveals putative genes involved in lycopodium alkaloid biosynthesis and developmental regulation
Source: BMC Plant Biol. 2010 Sep 21;10:209. doi: 10.1186/1471-2229-10-209 (PMC2956558; doi:10.1186/1471-2229-10-209)
Supplement: Additional file 4 — Validation of the SSR-containing unique putative transcripts (including contigs and singletons) by PCR amplification and Sanger sequencing. Unique putative transcripts chosen from the H. serrata and P. carinatus 454-EST dataset, including five singletons and five contigs for each species. Successful amplifications of these sequences and detections of SSRs are marked with "+" or "√", respectively; unsuccessful amplifications and detections are marked as "-". The primers used to amplify the sequences are also listed. The contigs were represented by Hs or Pc plused five digits and the singletons were represented by Pc plused 14 letters. [file 1471-2229-10-209-S4.DOC]

**Table S2: Validation of SSR-containing unique putative transcripts including contigs and singletons using PCR amplification and Sanger sequencing**

| **Unique putative transcript ID**^a^ | **Primer** | **PCR** | **SSR detection** |
| --- | --- | --- | --- |
| Hs00308 | **F:** TTGATTGGGAGGTCTTACT  **R:** GATATTCCGCTATGCGTTAT | **+** | **√** |
| Hs09760 | **F:** CGTAGAGGAGTATGAGGTG  **R:** TAGAGACAAGTGAGAGCAT | **+** | **√** |
| Hs06868 | **F:** GCATTAGACACAACAACTG  **R:** CGCTTGGCATACTTCTACT | **+** | **√** |
| Hs05747 | **F:** ACAAACTCACACTCACCTACT  **R:** ATCACTGAGCAAATAGTCCTG | **+** | **√** |
| Hs03951 | **F:** AGACAAGTCCCTCTCAAACAC  **R:** GCTGAGGAAGATGAGTTTGAT | **+** | **√** |
| HsFW1NBNE04I2UCY | **F:** ACAACACCAGGCAGACTAAAT  **R:** GACTGAGGAATAAACAAAGGC | **+** | **√** |
| HsFW1NBNE04JGTZO | **F:** GCAAAAACCTTGAACGAGAAC  **R:** GTTTCTGAACAAAGAGTGATG | **_** | **_** |
| HsFW1NBNE04JRP57 | **F:** ATCAGAAACTGTCAATGGTCG  **R:** CTCTCCACAATGATAGCCCAC | **_** | **_** |
| HsFW1NBNE04JPZML | **F:** ATGGCTTACGCTATCGGCTTG  **R:** CTTCACTTCTATCTACCTCAC | **+** | **√** |
| HsFW1NBNE04IQFLH | **F:** AACATTGTCGGCGATGGAGGC  **R:** CTCCCATTCTTCAAATACTTA | **+** | **√** |
| Pc04524 | **F:** CACACGGGTGGATTGCGGTTG  **R:** GTTTGTATTCACAGGGATTGG | **+** | **√** |
| Pc01174 | **F:** TGCCTCCTCCACCTCTCAAAC  **R:** CCTTTATTGCCCAAACTCTGA | **_** | **_** |
| Pc04808 | **F:** ATTTCTTTGGTTAGTTTCTGC  **R:** GCAACTCCAGCACCAACTATT | **+** | **√** |
| Pc01585 | **F:** ATCTCGTAGCAAAAGAACTGG  **R:** AGAAGGGGGAAGGCGGGGAG | **+** | **√** |
| Pc04810 | **F:** GGAGGACTTCAGCAGCAATG  **R:** GCCCCCTTGGGAAAACTGTG | **+** | **√** |
| PcFXAT9O007IKMGZ | **F:** CTCTCCCAGCGATTGCCG  **R:** GCTGCCACATCCTCTCAA | **+** | **_** |
| PcFXAT9O007HXDK2 | **F:** TAGAGATGTTACGCCGCA  **R:** CGCTCGTCGGTAGCAAGT | **+** | **_** |
| PcFXAT9O007H1FKH | **F:** ACGATGTTGTCCTGGAATGG  **R:** GTATTCTTTTAGTTACTTCTTTAT | **_** | **_** |
| PcFXAT9O007H4KVS | **F:** AAGTTCCCGAATTTTTGTATC  **R:** ACGAGCATGTTCTCCTCTTAG | **+** | **√** |
| PcFXAT9O007H72LU | **F:** CGAGTTGACAGGTGTTGATAG  **R:** CCAGATATTGGTTAGTAGCAC | **+** | **√** |

^a^Hs: unique putative transcripts from *H.serrata*; Pc: unique putative transcripts from *P. carinatus*.
