# Supplementary material for: Comparison of 454-ESTs from Huperzia serrata and Phlegmariurus carinatus reveals putative genes involved in lycopodium alkaloid biosynthesis and developmental regulation
Source: BMC Plant Biol. 2010 Sep 21;10:209. doi: 10.1186/1471-2229-10-209 (PMC2956558; doi:10.1186/1471-2229-10-209)
Supplement: Additional file 5 — Proposed biosynthetic pathways for Hup A and related lycopodium alkaloids in Huperziaceae plants. Proposed biosynthetic pathways for Hup A and related lycopodium alkaloids (From Ma and Gang, 2004). A. Proposed biosynthetic pathways for the precursors pelletierine and 4PAA. B. Proposed biosynthetic pathways from pelletierine and 4PAA to Hup A and related lycopodium alkaloids. [file 1471-2229-10-209-S5.DOC]

**Additional file 5**

**Proposed biosynthetic pathways to Hup A and related lycopodium alkaloids in *Huperziaceae* plants (From Ma and Gang, 2004).**

A. The proposed biosynthetic pathways to the precursors of pelletierine and 4PAA.

B. The proposed biosynthetic pathways from pelletierine and 4PAA to Hup A and related lycopodium alkaloids.


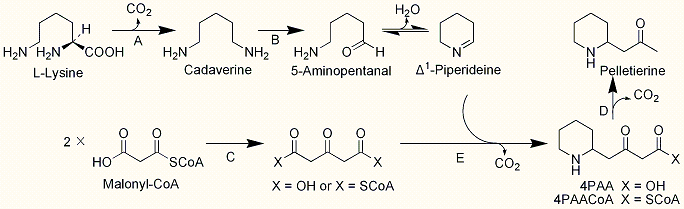


A


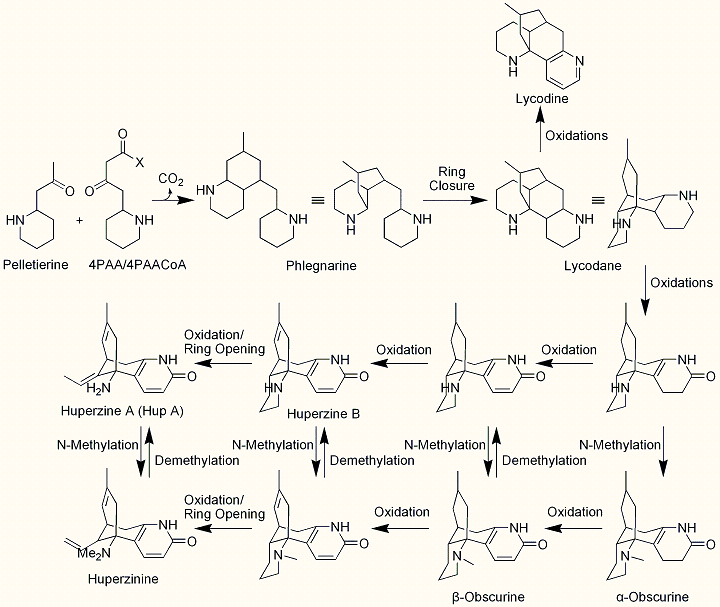


B
