# Supplementary material for: Comparison of 454-ESTs from Huperzia serrata and Phlegmariurus carinatus reveals putative genes involved in lycopodium alkaloid biosynthesis and developmental regulation
Source: BMC Plant Biol. 2010 Sep 21;10:209. doi: 10.1186/1471-2229-10-209 (PMC2956558; doi:10.1186/1471-2229-10-209)
Supplement: Additional file 9 — Summary of the CYP subfamily in the H. serrata and P. carinatus 454-EST database. The number of unique putative transcripts encoding putative CYP450s from H. serrata and P. carinatus belonging to different subfamilies. [file 1471-2229-10-209-S9.DOC]

## *Table S3: Summary of the CYP family in the H. serrata and P. carinatus 454-EST database*

| **CYP** | ***H. serrata*** | | ***P. carinatus*** | |
| --- | --- | --- | --- | --- |
|  | **Gene** | **Nos.^a^** | **Gene** | **Nos.^a^** |
| CYP6 | - | 0 | A1 | 1 |
| CYP51 | CYP51 | 3 | CYP51 | 1 |
| CYP71 | A6, A9, C3, D9 | 4 | A1,B34,B35,E1 | 7 |
| CYP72 | A1 | 6 | A1 | 5 |
| CYP73 | A1,A4,A10, A11,A14 | 6 | A1,A4,A11,A12,A13,A16 | 7 |
| CYP74 | - | 2 | - | 7 |
| CYP75 | A5,B1,B2 | 4 | A6,B1,B2 | 7 |
| CYP76 | C1 | 1 | C1 | 1 |
| CYP77 | A1,A2,A3 | 4 | A1,A2,A3 | 3 |
| CYP78 | A1 | 1 | A4 | 1 |
| CYP82 | A1 | 1 | - | 0 |
| CYP85 | - | 0 | A1 | 1 |
| CYP86 | A1 | 1 | - | 0 |
| CYP88 | A1 | 2 | - | 0 |
| CYP90 | A1,C1 | 10 | A1 | 2 |
| CYP93 | A3 | 1 | A2 | 1 |
| CYP94 | A2 | 1 | A2 | 1 |
| CYP97 | B2 | 3 | A2,B2,A3 | 5 |
| CYP98 | A1,A2,A3 | 6 | A1,A2,A3 | 4 |
| CYP524 | A1 | 1 | - | 0 |
| CYP704 | C1 | 5 | C1 | 6 |
| CYP707 | A1,A2,A3,A4,A7 | 11 | A1,A2,A3,A4,A7 | 8 |
| CYP716 | B1,B2 | 5 | B1,B2 | 5 |
| CYP720 | B1 | 1 | B2 | 1 |
| CYP725 | A2 | 2 | - | 0 |
| CYP750 | A1 | 6 | A1 | 2 |
| CYPCA | - | 1 | - | 1 |
| Others | - | 8 | - | 5 |
| **Total** |  | **96** |  | **82** |

^a^The numbers of unique putative transcripts with homology to cytochrome P450s.
