# Supplementary material for: Comparison of 454-ESTs from Huperzia serrata and Phlegmariurus carinatus reveals putative genes involved in lycopodium alkaloid biosynthesis and developmental regulation
Source: BMC Plant Biol. 2010 Sep 21;10:209. doi: 10.1186/1471-2229-10-209 (PMC2956558; doi:10.1186/1471-2229-10-209)
Supplement: Additional file 10 — Unique putative transcripts encoding putative CYP450s with sequence similarity between H. serrata and P. Carinatus. List of H. serrata contigs and singletons encoding putative CYP450s showing sequence similarity to Ph. carinatus unique putative transcripts. [file 1471-2229-10-209-S10.doc]

## *Table S4: List of the contigs of H. serrata encoding putative CYP450s showed sequence similarities to P. carinatus unique putative transcripts*

| **Group** | **Contigs of *H. serrata*** | **Contigs of *P. carinatus*** |
| --- | --- | --- |
| 1 | Hs01891 | Pc00236 |
| 2 | Hs09101 | Pc02140 |
| 3 | Hs12105 | Pc05169 |
| 4 | Hs08847 | Pc05572 |
| 5 | Hs11929 | Pc07642 |
| 6 | Hs11298 | Pc08305 |
| 7 | Hs02648 | Pc01388 |
| 8 | Hs05747 | Pc03281 |
| 9 | Hs12073 | PcFXAT9O007HYVX4 |
| 10 | Hs09894 | PcFXAT9O007IQ8BF |
| 11 | Hs01578 | Pc00994 Pc01787 |
| 12 | Hs06203 | Pc07664 Pc02973 |
|  | Hs07137 | Pc08941 PcFXAT9O007IQZDI |
| 13 | Hs00358 | Pc00876 PcFXAT9O007IF2ZL  PcFXAT9O007IR0DU |
| 14 | Hs02630  Hs02629 | PcFXAT9O007H2KC2 PcFXAT9O007H1FKH |
| 15 | Hs05836  Hs02656 | PcFXAT9O007IKQJI PcFXAT9O007II7SO |
| 16 | Hs00512 Hs04530 | Pc05423 PcFXAT9O007II5CJ |
| 17 | Hs00235 Hs00232 Hs00234 | PcFXAT9O007H441N |
| 18 | Hs02360 Hs02365 Hs02366 | Pc00275 |
| 19 | Hs13486 Hs13777  Hs13557 | Pc03772 Pc03770 Pc03771 |
| 20 | Hs04555 Hs06868 Hs10383 | Pc04975 Pc03343 |
| 21 | Hs03504 Hs03494 Hs03506 | Pc02293 PcFXAT9O007H3HNL |
| 22 | Hs02507 Hs00786 Hs00784  Hs00781 | Pc00909 |
| 23 | Hs00519 Hs12988 | Pc00477 Pc00485 Pc09110  Pc00460 Pc00462 |
| 24 | Hs00257 | Pc02200 Pc02201 Pc02204 |
| 25 | Hs06232 | Pc03842 Pc03841 Pc09105  Pc03840 |
| 26 | Hs09369 | Pc00300 Pc03153 PcFXAT9O007IF3FE |
| 27 | Hs01395 | PcFXAT9O007IH9IC PcFXAT9O007H3929 PcFXAT9O007IDG6T |
| 28 | Hs06334 | Pc02600 Pc07243 Pc02601 Pc02603  Pc02606 |
