# Supplementary material for: Comparison of 454-ESTs from Huperzia serrata and Phlegmariurus carinatus reveals putative genes involved in lycopodium alkaloid biosynthesis and developmental regulation
Source: BMC Plant Biol. 2010 Sep 21;10:209. doi: 10.1186/1471-2229-10-209 (PMC2956558; doi:10.1186/1471-2229-10-209)
Supplement: Additional file 12 — Major transcription factor families identified from H. serrata and P. carinatus using Inter-Pro. Unique putative transcripts from H. serrata (Sheet 1) and P. carinatus (Sheet 2) with similarities to genes encoding transcription factors. [file 1471-2229-10-209-S12.doc]

## Table S5: Major transcription factor families identified from *H. serrata* and *P. carinatus* using Inter-Pro

| **Transcription factor**  **family descriptions** | **Inter-Pro**  **accession Nos.** | **No. of unique putative transcripts** | |
| --- | --- | --- | --- |
|  |  | *H. serrata* | *P. carinatus* |
| AUX/IAA | IPR003311  IPR011525 | 9  23 | 11  24 |
| ARF | IPR010525 | 17 | 19 |
| B3 | IPR003340 | 0 | 26 |
| Basic helix-loop-helix | IPR001092 | 31 | 35 |
| Basic leucine zipper (bZIP) | IPR011700  IPR004827  IPR011616 | 1  33  29 | 4  27  21 |
| Helix-loop-helix | IPR011598 | 29 | 35 |
| Helix-turn-helix | IPR000047 | 8 | 1 |
| Homeobox | IPR006455  IPR017970  IPR001356  IPR003106 | 4  26  43  0 | 4  24  35  2 |
| Homeodomain-like | IPR009057 | 92 | 79 |
| Homeodomain-related | IPR012287 | 82 | 71 |
| Myb | IPR015495  IPR014778  IPR017877  IPR006447  IPR017930  IPR016310 | 9  54  18  34  44  0 | 15  48  11  25  41  1 |
| NAC | IPR002715 | 0 | 2 |
| Pathogenesis-related | IPR001471 | 15 | 21 |
| WRKY | IPR003657  IPR017396  IPR018872 | 14  1  5 | 22  3  5 |
| Zinc finger, C2H2 | IPR015880  IPR013087  IPR007087  IPR003656 | 31  3  30  1 | 22  1  22  0 |
| Zinc finger, CCCH | IPR000571 | 21 | 20 |
| **Total No. of TFs** |  | **504** | **469** |
